# Ecological Momentary Assessment in Behavioral Research: Addressing Technological and Human Participant Challenges

**DOI:** 10.2196/jmir.7138

**Published:** 2017-03-15

**Authors:** Lora E Burke, Saul Shiffman, Edvin Music, Mindi A Styn, Andrea Kriska, Asim Smailagic, Daniel Siewiorek, Linda J Ewing, Eileen Chasens, Brian French, Juliet Mancino, Dara Mendez, Patrick Strollo, Stephen L Rathbun

**Affiliations:** ^1^ Department of Health & Community Systems University of Pittsburgh School of Nursing Pittsburgh, PA United States; ^2^ Department of Epidemiology University of Pittsburgh Graduate School of Public Health Pittsburgh, PA United States; ^3^ University of Pittsburgh School of Medicine Pittsburgh, PA United States; ^4^ Institute for Complex Engineered Systems Carnegie Mellon University Pittsburgh, PA United States; ^5^ Human Computer Interaction Institute Carnegie Mellon University Pittsburgh, PA United States; ^6^ Department of Epidemiology and Biostatistics University of Georgia College of Public Health Athens, GA United States

**Keywords:** ecological momentary assessment, relapse, obesity, smartphone, eating behavior, adherence

## Abstract

**Background:**

Ecological momentary assessment (EMA) assesses individuals’ current experiences, behaviors, and moods as they occur in real time and in their natural environment. EMA studies, particularly those of longer duration, are complex and require an infrastructure to support the data flow and monitoring of EMA completion.

**Objective:**

Our objective is to provide a practical guide to developing and implementing an EMA study, with a focus on the methods and logistics of conducting such a study.

**Methods:**

The EMPOWER study was a 12-month study that used EMA to examine the triggers of lapses and relapse following intentional weight loss. We report on several studies that informed the implementation of the EMPOWER study: (1) a series of pilot studies, (2) the EMPOWER study’s infrastructure, (3) training of study participants in use of smartphones and the EMA protocol and, (4) strategies used to enhance adherence to completing EMA surveys.

**Results:**

The study enrolled 151 adults and had 87.4% (132/151) retention rate at 12 months. Our learning experiences in the development of the infrastructure to support EMA assessments for the 12-month study spanned several topic areas. Included were the optimal frequency of EMA prompts to maximize data collection without overburdening participants; the timing and scheduling of EMA prompts; technological lessons to support a longitudinal study, such as proper communication between the Android smartphone, the Web server, and the database server; and use of a phone that provided access to the system’s functionality for EMA data collection to avoid loss of data and minimize the impact of loss of network connectivity. These were especially important in a 1-year study with participants who might travel. It also protected the data collection from any server-side failure. Regular monitoring of participants’ response to EMA prompts was critical, so we built in incentives to enhance completion of EMA surveys. During the first 6 months of the 12-month study interval, adherence to completing EMA surveys was high, with 88.3% (66,978/75,888) completion of random assessments and around 90% (23,411/25,929 and 23,343/26,010) completion of time-contingent assessments, despite the duration of EMA data collection and challenges with implementation.

**Conclusions:**

This work informed us of the necessary preliminary steps to plan and prepare a longitudinal study using smartphone technology and the critical elements to ensure participant engagement in the potentially burdensome protocol, which spanned 12 months. While this was a technology-supported and -programmed study, it required close oversight to ensure all elements were functioning correctly, particularly once human participants became involved.

## Introduction

Ecological momentary assessment (EMA) assesses individuals’ current experiences, behaviors, and moods as they occur in real time and in their real-world settings [1]. Studies employing EMA methods have become more common in recent years, partially spurred by the ubiquitous availability of mobile devices and wearable sensors that provide access to individuals in their natural environment. This growth in EMA studies has expanded the array and scope of behaviors being studied. However, there continues to be an emphasis on substance abuse, particularly smoking [2-4]; other areas of focus include chronic pain [5-7], physical activity [8,9], lapses among dieters [10,11], and eating behaviors [11-14].

While a typical study may assess behaviors and emotions by retrospective questionnaires, this approach misses the specific situations that precipitate behavior changes. Most behaviors are dynamic and may change frequently throughout the day depending on context and social setting. The benefit of using EMA in these studies is that, through frequent assessments of ongoing life circumstances, EMA permits the estimation of risk of antecedents to the occurrence of a specific behavior. Unlike instruments or methods that are abstracted from the context in which events occur, EMA is able to capture environmental influences in a direct and immediate manner, which provides a direct examination of the mechanisms linking the immediate environment with risk, and, with aggregation, can also capture broader processes that may undermine behavior change [10,15]. EMA has made significant contributions to the behavioral sciences by describing dynamic changes in behaviors across contexts and environments in the everyday lives of study participants. However, despite its numerous benefits and the enhancement of this method by the technology available today, the burden that real-time, longitudinal data capture can place on the participant and the research team is significant.

We are using our weight loss relapse study as an exemplar to describe how we approached the EMA study. Relapse and weight regain are major issues in the treatment of obesity [16], yet knowledge of how relapses occur following intentional weight loss is rather limited. This is, in part, because of the dearth of prospective studies in the literature and the methodological limitations of the few studies reported [17,18]. To ensure the reader has an understanding of the major constructs discussed in our study of weight loss and relapse, we provide an operational definition of the terms that are the focus in several studies using EMA: temptation, lapse, and relapse. A *temptation* is a desire or sudden urge to eat something that was not in the individual’s eating plan for that day or that would result in his or her exceeding the daily calorie or fat gram goal. A *lapse* means acting on that temptation and eating the food that fell outside the plan or goal, or that left a person feeling that he or she cheated on his or her diet. If the participant planned to eat a special dessert and budgeted his or her calories for this indulgence, it would not be a lapse, but if it occurred spontaneously without regard for their daily goal, it would be considered a lapse [11,19-21]. Lapses are contained, time-limited events. In contrast, a *relapse* is repeated episodes of lapsing, or what has been described as a return to previous behavior [19]. Marlatt and Gordon’s [22] cognitive behavioral model of relapse underscored the processes by which an initial lapse could lead to a full-blown relapse; for example, eating a calorie-dense meal or dessert could lead to the person returning to their previous eating behaviors. The individual’s cognitive and affective responses to a lapse are critical determining factors of whether the lapse will deteriorate into a relapse.

While the field of EMA studies has expanded rapidly, and the assessment of EMA has progressed from use of paper diaries to wireless devices, the literature is void of practical or procedural guidance on how to develop and implement an EMA study that assesses single or multiple behaviors over brief or extended periods. The purpose of this paper is to present practical steps and lessons learned in developing and implementing an EMA study, with a focus on the methods and logistics of conducting such a study. We also include strategies to ensure adequate adherence to EMA prompts; thus, we report data on adherence to daily EMA prompts and compare these data with those reported by studies of varying duration and frequency of EMA prompting. We include data from the EMPOWER study, which was a 12-month study that used EMA to examine the triggers of lapses and relapse following intentional weight loss. All participants were provided standard behavioral treatment for weight loss, which provided the background for the study of relapse. Standard behavioral treatment includes lifestyle modification, an approach that includes reduced energy intake, increased energy expenditure, and behavioral change strategies taught and practiced in groups [23]. The core behavioral change strategies are based on social cognitive theory and include goal setting, self-monitoring, cognitive restructuring, self-efficacy enhancement, and social support with feedback and guidance provided by interventionists to assist with development of problem-solving skills [23-28]. We prompted participants daily to complete EMA surveys at the beginning of the day (BOD) and end of the day (EOD) and also at random times during waking hours.

## Methods

Given the theoretical and methodological reasons to collect EMA data using mobile devices, collection in this manner raises considerable practical considerations and challenges. The following section includes lessons learned from previous studies, which were extremely important in informing the development of the EMPOWER study.

### Part 1: Steps to Develop and Implement an EMA Study and Its Supporting Infrastructure

#### Defining an EMA Daily Data Collection Protocol

EMA employs 3 types of data collection protocols: *even* *t* *co* *ntingent*; *si* *gnal* *co* *ntingent* or “ *ra* *ndom*”; and *tim* *e* *co* *ntingent* [29]. The EMPOWER study used all 3 types of data collection protocols.

Individuals were instructed to initiate an *ev* *ent-* *contingent* entry in the smartphone-based EMA app when some predefined event had occurred, such as a strong temptation to overeat, or a lapse (eg, having acted on a temptation and eaten some food inconsistent with a planned diet).

We used *signal-contingent* assessments scheduled at random times to obtain a representative sample of participants’ moods and environments over the course of their study participation. These are “signal-contingent” assessments because the participant responds to a signal delivered at random, such as a beep from the device. Since signal-contingent assessments were delivered according to a known probability-based sampling design, we aggregated data to obtain unbiased estimates of the mean levels of the individuals’ moods or levels of energy throughout the duration of the study. Moreover, we combined information from event-contingent assessments with that from the signal-contingent assessments to estimate risk of a salient event as a function of antecedents to the events, such as mood and environment [1,30,31].

*Ti* *me-* *contingent* assessment prompted the individual to make an entry at a fixed time, such as the beginning of each day, to assess the previous night’s sleep. This process can be described as sampling a data collection event. Each of the 3 types of data collection protocols captures a data point on a target behavior, but any can be further sampled, such as additional questions posed regarding emotional state or environmental context. We provide more detail on architecting the assessment questions and response options, as part of finalizing the EMA protocol in the section on pilot studies.

#### Preliminary Studies With Electronic Diaries

Whatever the theoretical and methodological reasons to collect EMA data and to use mobile electronic devices to do so, such studies raise considerable practical considerations and challenges. The EMPOWER study was built on what our team learned from a clinical trial and pilot studies testing various devices and EMA sampling strategies. From 2004 to 2009, we conducted a 24-month clinical trial (Self-Monitoring And Recording using Technology [SMART]) that used personal digital assistants (PDAs), which required carrying the PDA and a cell phone [32]. We learned that carrying only 1 device was important to most people and that people had difficulty keeping 2 devices charged. Thus, our previous experience with technology and conducting 3 pilot studies testing various phones and EMA sampling strategies was extremely important in facilitating the success of the EMPOWER study.

#### Pilot Study 1: Test of Basic Infrastructure

The data collection architecture was a 3-tiered design with distributed Android (Google, Mountain View, CA, USA) app clients communicating with a public-facing Web server backed by an Oracle database (Oracle Corporation, Redwood Shores, CA, USA). To ensure that all pieces of technology (ie, smartphone, server, and database) worked in a synchronous and efficient way, we conducted thorough in-house testing. Research and management staff with varied technology skills and experiences used different makes and models of Android smartphones to identify as many bugs as possible related to differences in the Android operating system versions. We tested all aspects of end-to-end data flow, including sending interview data to the database, checking for updates to study scheduling parameters, and checking for app updates to resolve bugs. We also tested the functioning of the EMA algorithm focused mainly on correctness of the scheduling of EMA events; that is, that events were scheduled at the correct times and that the scheduled events actually occurred when intended.

Lessons learned: After a thorough 2-week in-house test, we learned more about the infrastructure needs to support data flow. In particular, we added reporting of more phone side events, such as phone restarts, and scheduled times of interviews to provide a more complete picture of the participants’ interactions with the phones to aid in identifying when error conditions (such as scheduled interviews that never fired) occur. The multiple phone model testing allowed us to resolve a few model-specific bugs in the EMA app, but also let us identify which phone manufacturers and phone models behaved the most reliably. As many study participants had to upgrade to a compatible Android phone to join the study, we recommended they upgrade to one of the models that we found to be the most reliable, if possible [33].

#### Pilot Study 2: Test of EMA on Different Phone Models

We conducted a small pilot study with former participants (N=16) in weight loss studies conducted in our laboratory [34,35]. The purpose of the study was to evaluate acceptability and feasibility of using smartphones to collect EMA data. We provided the participants with a smartphone (Android; or iOS, Apple Inc, Cupertino, CA, USA) and training session on how to use the phone. We programmed the smartphones to prompt the participants 6 times per day at random times between 8:00 AM and 9:00 PM, and the participants were instructed to initiate a survey if they experienced a strong temptation to “go off their eating plan,” or if they acted on this temptation and had a lapse.

Lessons learned: Initially, we sampled individuals 8 times per day with EMA prompts and received feedback from 10 of the 16 participants that this was too intensive. They felt that no more than 5 times per day was acceptable. Therefore, we reduced the prompts to 5 per day. We sampled participants on average for 21 days. Response to random prompts was approximately 71% and to end-of-day prompts, approximately 55%.

Acceptability: Overall, 100% of the participants strongly agreed or agreed that it was easy to use the smartphone; 75% (12/16) strongly agreed that 5 EMA prompts per day were adequate, and 69% (11/16) said they would consider participating in a study lasting 6-12 months.

Lessons related to iOS: Since we did not have access to the full functionality of the iOS, we could not wake the phone and schedule EMA prompts; thus, the app was always running in the background and quickly drained the battery power. We learned that using phones with the iOS operating system required us to provide participants with 2 chargers, as the person needed to charge the phone during the day. In this pilot phase, we used first-generation iOS phones; however, because we did not have access to the operating system codes beyond the first generation of these phones, we could not program the EMA on later generations of this phone [36]. The Android operating system proved more accommodating; however, this may not be an issue today if the EMA programming is done on the server side.

Lessons learned related to personal use of phones: Overall, the pilot sample of 16 and the longer study period of 21 days permitted us to improve our backend systems to capture new issues more efficiently and act upon them in a timely manner, such as the impact of using other features or apps on the phone. We provided participants’ phones with all features available for their use, unlike some studies that provided phones with everything locked except the EMA features. Participants tested the EMA app using study-provided phones, which helped us refine the questions and delivery of prompts, and also reinforced the notion that people who did not regularly use their mobile phones were more likely to forget about them, thus leaving them at home or letting the battery drain. Thus, we screened individuals for being a user of a mobile phone.

#### Pilot Study 3: Testing and Refining EMA Items

We conducted a pilot study through an anonymous Web-based questionnaire (N=133) with individuals who had previously participated in weight loss studies conducted in our laboratory [37]. The purpose of the study was to test and refine the item content for 4 types of EMA surveys: event-contingent, signal-contingent, and beginning-of-day and end-of-day surveys. We presented participants with draft assessments, and they provided feedback on the clarity of the instructions and assessment items.

**Figure 1:**
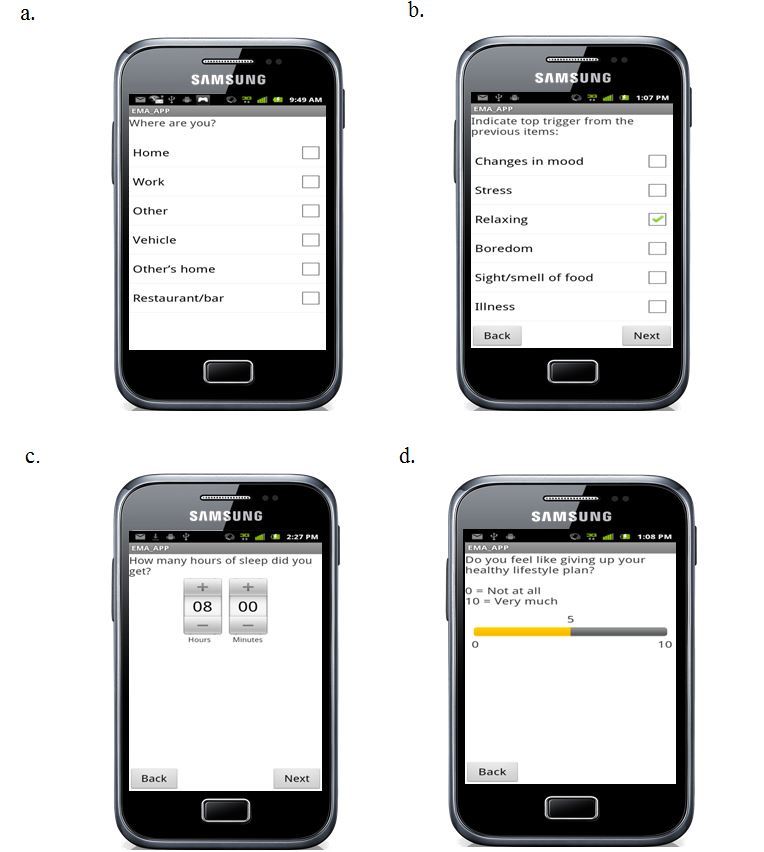
Sample screenshots of ecological momentary assessment questions: (a) randomly scheduled, (b) self-initiated, (c) beginning of day, (d) end of day.

Lessons related to testing prompts and refining EMA items: Participants thought the EMA items were acceptable but expressed concern about the volume of prompts they received and how to enter data to complete the EMA assessments. We learned that it was essential to train participants how to adequately complete the prompts and manage signals, such as using snooze mode to delay a reply. Figure 1 shows screenshots of EMA items.

#### Summary of Pilot Studies

We learned a great deal in the series of pilot studies; most salient was the issue of the restrictive nature of Apple Inc to vet an app into iTunes. Thus, we decided not to pursue programming the EMA app on devices that had iOS. Concurrently, the Android-based smartphone was gaining in acceptability and, thus, we chose to use this model for the EMA programming. It also became clear that, regardless of the individual’s technology expertise or skill, all participants would need training in the various aspects of providing data via a smartphone.

### Part 2: EMPOWER Study: Applying the Lessons Learned in Developing the Infrastructure

**Figure 2:**
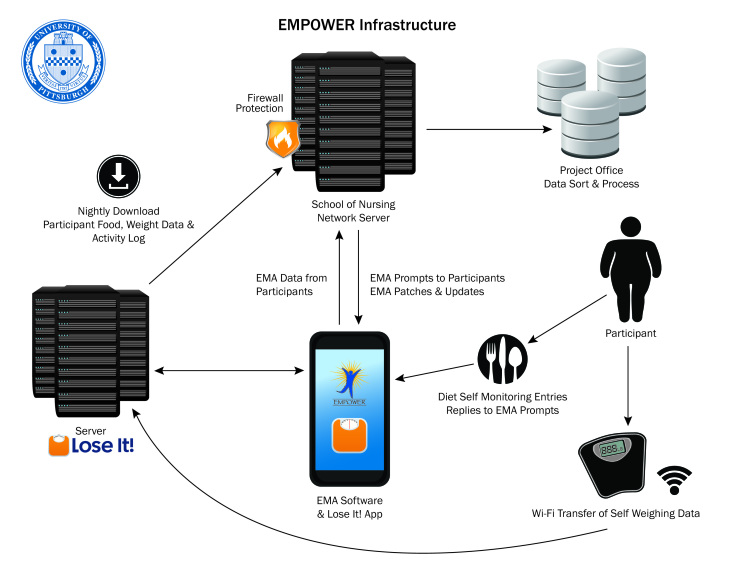
EMPOWER study infrastructure supporting collection of self-monitoring, weight, and ecological momentary assessment (EMA) data.

The series of studies and lessons described above resulted in our developing the infrastructure that supported the EMPOWER study. The EMA data collection system comprised 3 primary components: an Android smartphone, a Web server, and a database server (Figure 2).

To support real-time data collection using an Android-based app, Wi–Fi-enabled weight scales, and self-monitoring third-party data through a commercially available self-monitoring app (Lose It!, FitNow, Inc, Boston, MA, USA), we went through the following steps in the development process: (1) determined the data flow to and from all sources, (2) identified storage and server processing solutions to support the data flow, (3) developed EMA survey apps and determined how to deliver updates to smartphones in real time, (4) acquired server solutions from the University of Pittsburgh and access to third-party data (Lose It!), (5) conducted thorough internal and pilot testing, and (6) developed proper oversight and troubleshooting plans for day-to-day operations and monitoring of the system.

#### Data Flow

It was critical for us to define the data flow from all sources that would later drive decisions needed on the number and type of servers. We identified that the data would come from 2 sources: participants’ smartphones and the Lose It! servers that contained self-monitoring and weight data synchronized with participants’ Lose It! account. Communication between servers and smartphones needed to be bidirectional so that the EMA app could be updated or patches released, as well as for downloading data from the smartphones and Lose It!. A thorough needs assessment demonstrated that we would need a Web server to process the data and a database server to store data. The administrative staff of FitNow, Inc agreed to have the self-monitoring data for the study participants downloaded from a special portal to our university server every night at midnight. We did this with a 24-hour delay so that if a participant did not record their dinner until the next morning, the data would be included in the download.

#### Web Server

The Web server was composed of scripts that were either called directly by the smartphone app with scripts or internally initiated data downloads on a scheduled basis. The smartphone scripts were called periodically by the participants’ smartphones to send new survey and event data, or to retrieve updates for the app. Internally scripts were called once a day to collect calorie, exercise, and weight data from Lose It! and to verify that each participant received each of the scheduled EMA surveys.

#### Database Server

We used an Oracle server (version 10g, Oracle Corporation) as the backend database server. This relational database served as a repository for all EMA, Lose It!, and weight data, as well as a large amount of processing data. Each EMA assessment included a date and time stamp when the EMA interview was delivered, when a participant started and ended it, and its status (completed, missed, or abandoned).

#### Smartphone EMA Survey App

The smartphone EMA app developed for this study was based on an existing app that had been developed for another research group [38,39]. The pre-EMPOWER version of the app was designed to work as a stand-alone research tool with study-managed, locked-down Android devices. Android phones were chosen as the target technology for this app because they supported the full range of control the phone needed to support the study. These control points included reliable scheduling of EMA assessments, which was difficult to implement in iOS at the time our study was conducted. However, this specific problem has been resolved, as programming can be done on the server side instead of on the phone today, thus permitting use of different operating systems. Still, operating system compatibility is a technical concern that should always be considered early in development. To meet the study’s requirements, the app had to be made robust enough to enable long-term running on participant-owned and -managed phones. Even so, having the existing infrastructure of the pre-EMPOWER app greatly reduced the development time needed to reach a stable, pilot-testable version of the EMPOWER EMA app.

Each participant had a copy of the app installed on their own smartphone and configured for the participant. The app provided most of the functionality of the system, including (1) scheduling all types of interview prompts (ie, BOD, which was set by the participant depending on when she or he woke up; EOD was also set by the participant and had to be at least 12 hours after BOD; and random), (2) providing the appropriate version of survey content as needed, and (3) transmitting recorded survey responses and important events (eg, survey scheduling, phone reboots, and update install times) at regular intervals to the Web server that logged these to the database, and polling the Web server for updates in parameters, any patches, and possible new EMA versions. Data were stored locally on the phone regardless and kept until transmitted to the server even if a few tries were required.

We decided to place the majority of the system’s functionality for EMA data collection on the participants’ phones to avoid loss of data when there was no network connectivity. Data would be transmitted to the server when connectivity was restored. This was especially relevant for a study with a 1-year duration. Participants were likely to have loss of network connectivity for hours (such as in certain campus buildings or travelling through rural areas) to days at a time (such as with travel on vacation or business). It also protected the data collection from any server-side failure or down time. By distributing the EMA data collection functionality among the participants’ devices, no single system component failure could negatively affect data collection across all participants.

#### Access to Servers

The University of Pittsburgh’s enterprise Web infrastructure offered a robust server hosting the university's community and research studies, which was the perfect fit for the study. After negotiation and ensuring the enterprise Web infrastructure programmers that the smartphones would not be using excessive bandwidth and that no sensitive or confidential information would pass between phones and servers, we gained access to the PHP (a server-side scripting language) Web server and Oracle database server described above. Every phone/participant had a unique ID, and all responses to questions were either integers or, in a few cases, free text, where participants had to further explain their selection. To access self-monitoring and weight data through Lose It!, we negotiated special access to those data for download to Oracle servers and accessed the data via a custom-made management portal for study interventionists to review participants’ data through the Lose It! Web interface.

#### Day-to-Day Operations

With all participants in the field and with real-time data collection for 12 months, it was critical for us to ensure that participants were receiving EMA prompts when they were scheduled. To achieve this, we collected additional data to help us minimize down time in the case of a smartphone malfunctioning. Those data were stored in our database and consisted of all scheduled prompts recorded in a table along with boot and shutdown times when participants rebooted their phones. Every day, we ran a custom report that documented the last time a prompt was fired and when the next prompt was going to occur. If someone was scheduled to receive a prompt but did not, an email was sent to that participant to investigate if anything was wrong with the phone, if the app was still installed, or if there were problems with the Internet connection, such as no connection or a weak one. We found that, even if there was no Internet connection, participants were still receiving prompts and the data were queued for upload at the next connection to the Internet.

#### Data Management and Data Security

EMA data collected by smartphones were first processed by a Web server and then transferred to a secure database server in real time. The database and Web servers were hosted and maintained by the University of Pittsburgh Computing Services and Systems Development Network Operations Center that has 24/7 monitoring. The servers were behind the firewall with special permission given to us to pass the data. Security was reviewed by the university before we were granted permission to build the infrastructure. The dietary self-monitoring data were maintained on the Lose It! server by Fit, Inc. Overall, no personal health data were ever included in the data transmission. All participants were assigned random unique ID numbers and most of the data were integer values. The data were backed up nightly. While the servers were hosted and maintained by the University of Pittsburgh Network Operations Center, they were also overseen by the University of Pittsburgh School of Nursing’s database administrator.

### Part 3: Challenges Faced and Lessons Learned From the Conduct of the EMPOWER Study

The 1-year EMPOWER study infrastructure faced several challenges from which lessons may be learned regarding (1) variation among mobile phone models, manufacturers of the operating systems, and different versions of the same Android operating system, (2) phone operating system updates, and (3) conflict with nonstudy use of mobile phones. Previous experience and the study’s 12-month duration drove the decision to use participants’ personal smartphones for data collection but introduced the technical challenge of supporting a wide range of smartphone models and operating system versions. While all of the Android smartphones broadly used the same operating system, and Android claims interoperability, there were significant and subtle differences in operating system behavior between smartphone manufacturers and also between operating system versions from a single manufacturer.

#### Phone Operating System Updates

In at least two cases, carrier-released operating system updates led to failures in the smartphone app that caused a loss of data. In both cases, the updates changed underlying operating system resources that caused the app to crash when attempting to prompt the user to complete a survey. In both cases, the operating system update-related error was only resolved after uninstalling and reinstalling all affected software. Tracking changes in the operating system update events on the participant’s phone reduced the time to identify this potential source of failure. To address these issues, we recommend, in addition to automating tasks as much as possible to shorten troubleshooting turnaround time, having a system in place that collects useful data (eg, technical and user behavior) to stay informed. We also recommend doing thorough user training, always listening and responding to end users’ observations, and collecting information on problems encountered. Each of these activities was helpful in addressing technical issues.

#### Conflict With Nonstudy Use of the Smartphone

The use of participants’ personal smartphones for data collection introduced an additional source of variability that could lead to a range of problems. Nonstudy-related uses of the smartphone could affect the ability of the EMA app to function as intended, such as turning off the smartphone, and interactions with other installed apps led to scheduled EMA assessments failing to launch and prompts being missed. By looking at logs and talking to the participants, we could try to pinpoint what led to problems in the system; we could then educate the participants on how to avoid such problems.

### Institutional Review Board: Preparing for Institutional Review Board Approval Today

This study was approved by the University of Pittsburgh institutional review board in 2010 without any concerns except the risk for participant burden related to use of the EMA or the dietary self-monitoring app. However, recently the institutional review board has increased its scrutiny of protocols using mobile devices. For protocols that use any form of apps since 2016, the investigator is required to answer an extensive list of questions and document details regarding the risk of a third party intercepting data, data plan expenses, data security, terms of agreement of the commercial app, and concern if a mobile app is deemed a mobile medical app, in which US Food and Drug Administration regulations apply. Investigators need to provide the answers to these concerns as well as the personnel to arrange for data exchange and security.

### Training Participants in EMA and Ensuring Adherence to EMA Protocol

We used a single-group, observational study design to describe the microprocesses of lapse and relapse following intentional weight loss through the use of daily EMA surveys over 12 months. The 12-month duration was based on extensive data showing that individuals usually reach their peak weight loss at 6 months, which is often followed by partial or total regain of the weight that had been lost [40]. To provide the background for weight loss and regain, the study implemented a standard behavioral intervention for weight loss that was implemented through group sessions over the 12 months. The following paragraphs focus on the details of the EMPOWER study pertinent to the EMA protocol, such as training study participants in the use of a smartphone and completing the EMA data sampling, as well as strategies to enhance adherence to the EMA survey completion. Details of standard behavioral interventions for weight loss conducted in our laboratory are published elsewhere [32,35,41].

Once we verified that individuals met the standard eligibility criteria for behavioral treatment for weight loss, we confirmed whether they could participate in a study that required their response several times a day. This included a trained research staff person asking the person about their daily routine, the demands of their job, and whether they were permitted to have their phone nearby in the work environment. The staff person walked through the daily routine of EMA prompts and demonstrated the surveys on a phone and how to reply. Thus, potential participants were fully informed of the demands of the study and were told that they were vital partners in this research on identifying the triggers for relapse in real time. The innovative components of the study seemed to inspire some to want to participate and remain in the study. Developing this partnership at baseline and throughout the study and talking to participants in the treatment group sessions periodically about what we were aiming to learn in the study likely helped ensure the importance of their contributing. We have a track record of good retention in clinical trials, so we used standard strategies such as following up with an email or phone call if a person did not attend a treatment session or if we did not hear from them, or if we were not seeing self-monitoring data. We tried to be as flexible as possible within the limitations of a study protocol. However, while we may have permitted a participant to miss some sessions, we required participants to complete at least 60% of the daily EMA surveys to receive reimbursement for the data plan charges.

After determining participants’ willingness to engage in the daily EMA surveys, we confirmed whether the individuals had a study-compatible smartphone. If they did not, we gave them written instructions on which phone to purchase and to bring in the receipt so the study could reimburse them for the expense. Once they had the phone, they were scheduled for a one-on-one enrollment session, during which the EMA app was installed on their phone and they were shown how to complete the EMA surveys and were required to give a return demonstration completing a survey to the staff person. As Figure 1 shows, the survey items were on the home screen of the phone, and replies were indicated by touching the screen or sliding a bar for a Likert-scale response. Printed instructions with illustrations were also provided. If participants needed additional assistance, a staff member was available to troubleshoot by phone or in person. Participants were also provided with a Withings Wi–Fi scale (Withings, Inc, Cambridge, MA, USA) for daily self-weighing and printed instructions on how to set it up in their home. The scale transmitted the weight to the Lose It! server, which was visible when participants accessed Lose It! on their phone. We had instant access to information regarding participants’ completion of EMA survey and use of the scale. If there appeared to be any problems, such as no weights being recorded or no responses to EMA prompts, the Data Manager alerted the interventionists, who contacted the participant to determine the problem and help resolve it. To ensure that participants knew what the terms *temptation*, *lapse*, and *relapse* meant for this study, we explained these terms as described above and illustrated them with several practical examples. We had several discussions with the participants about these terms and their related meanings and clarified the definitions as necessary until the participants were able to articulate the terms and definitions in their own words.

### Incentives to Enhance Adherence to EMA

The pilot study increased our awareness of the risk of participant overburden inherent in the EMA study. To encourage responding to as many prompts as possible, we offered an incentive using a random, variable schedule based on a lottery principle, so that the more frequently the participants responded, the higher their likelihood of receiving an incentive (eg, a US $5.00 gift card). Participants also were told that they needed to complete 60% of the random prompt surveys in order to receive US $25 each month to compensate for a portion of the data plan fee. Others have used financial incentives for EMA completion and reported that it enhanced compliance [12]. Anecdotally, many of our participants reported that it did not matter, as they had family shared data plans, and some did not collect their incentives.

### EMA Monitoring

Our EMA monitoring protocol was adapted from earlier studies conducted by Shiffman and colleagues [38,42]. Participants were instructed that they would be monitored for the duration of the 12-month study and that the frequency of prompts they received on their smartphones would vary over time. At random times throughout the waking hours of the day, the smartphone prompted the participants with an alarm tone to answer the EMA survey questions. If the individual was unable to respond immediately (eg, he or she was in a meeting), he or she could delay answering the prompt for up to 20 minutes, which was done by using a snooze function on the alarm for 5 minutes up to 4 times. The participant also could temporarily turn off the alarm tone when it would be disruptive (eg, sleeping, in church, or attending a performance). The number of items per prompted assessment varied, as did the questions, but there were usually 10-15 items, with some questions being asked every time. Questions and the response options were displayed on the screen; the app permitted touch screen responses to complete the assessment (see Figure 1). Skip patterns were used whenever appropriate to reduce participant burden. For example, participants could not observe others in their immediate environment eating if they were completely alone, and thus were not asked that question. As described earlier, there were 4 types of EMA prompting. Both the BOD and EOD surveys were delivered at a time indicated by the participant as acceptable. Use of the alarm mode on the smartphone also served to set limits on the times that random prompts could be delivered, but participants were required to have at least 12 hours between morning waking and going to sleep.

### Duration of the Study

The typical weight change pattern observed in several reported studies revealed that weight loss often reaches its peak at approximately 6 months after initiation of treatment, followed by a 30% to 35% weight regain [43-45]. One of our previous studies demonstrated that 52.4% of the participants achieved significant weight loss at 6 months but began to regain in the months that followed [46]. Given these results, we selected 12 months of observation to capture the natural trajectory of weight loss and regain, specifically to capture in real time the triggers of lapses and relapses.

## Results

Throughout this paper, we have addressed the challenges we encountered in pilot testing the EMA surveys and the mobile technology to facilitate the transmission of prompts and responses providing data. Through the pilot studies and the 12-month observation study that collected daily EMA data, we learned many lessons. Textbox 1 summarizes those key lessons.

**Summary of lessons learned regarding collection of ecological momentary assessment (EMA) data.**

Participant-related preferences

- Carrying only 1 device was important to most people.
- People had difficulty keeping 2 devices charged.
- Random EMA prompts should be limited to a maximum of 5/day.
- Individuals who were not regular mobile phone users were not good candidates, as they might not keep a phone charged or nearby.
- Regardless of their technology experiences, it is essential to train all individuals in the use of a phone and in completing EMA prompts, and having staff available to assist with troubleshooting is advised.
- Providing the participant with a phone for their full use facilitated their keeping it charged and with them.
- To ensure compatibility with the needs of the study, a list of recommended phones was provided so that each participant could select their phone model.
- It is important to listen to end users to refine EMA questions and the assessment schedule.

Infrastructure-related needs

- Experience enabled the team to identify the most reliable phone models.
- The battery of phones with iOS drained quickly, requiring chargers at home and in the workplace.
- Phones with the Android operating system supported the full range of control needed to support the EMA study, permitted programming to be done on phone, rather than being Web based.
- To avoid loss of data due to loss of network connectivity, the majority of the system’s functionality for EMA data collection was placed on the participants’ phone (important for a person who travels).
- For a long-term study, it is important to track operating system updates that could potentially interfere with EMA; also, use of other apps installed by the participant might interfere with EMA prompts.

### EMPOWER Study Sample

We completed enrollment in January 2014 with a sample of 151 enrolled over 2 years in 6 cohorts. No EMA data were collected from 1 participant who withdrew from the study immediately after baseline; data from this participant were not included in the following analyses. The sample (see Table 1) was predominantly female (136/150, 90.7%), white (121/150, 80.7%), employed full-time (124/150, 82.7%) and well educated, having completed a mean of 16 years of education. Participants were, on average, 51.09 (SD 10.19) years of age. The mean body mass index was 34.02 (SD 4.58) kg/m^2^. The final cohort completed the study in March 2015 with an overall 87.4% retention rate. Reasons for participant withdrawal were pregnancy (n=3), development of diabetes (n=5), personal decision to withdraw (n=4), and lost to follow-up (n=7), resulting in a final sample of 132 participants (132/151, 87.4%) completing the final assessment.

**Table 1:** Sample characteristics at baseline (n=150).

Characteristics		n or mean	SD or %
Age in years, mean (SD)		51.09	10.19
Education in years, mean (SD)		16.41	2.81
Body mass index in kg/m^2^, mean (SD)		34.02	4.58
Female sex, n (%)		136	90.7
White racial status, n (%)		121	80.7
**Marital status, n (%)**			
	Married, or living with partner or significant other	93	62.0
	Never married	25	16.7
	Widowed, separated, or divorced	31	20.7
Employed full-time, n (%)		124	82.7
**Household income in US $, n (%)**			
	<50,000	35	23.3
	≥$50,000	107	71.3
**Have intentionally lost 10-19 lbs, n (%)**			
	Never	8	5.3
	1-2 times	60	40.0
	3-5 times	55	36.7
	6-10 times	17	11.3
**Have intentionally lost 20-49 lbs, n (%)**			
	Never	51	34.0
	1-2 times	70	46.7
	3-5 times	16	10.7

### Adherence to Answering and Completing the EMA Prompts

Table 2 presents the details of adherence by EMA prompt type. During the first 6 months of the 12-month study interval, participants completed 88.26% (66,978/75,888) of the daily random assessments and discontinued or abandoned less than 1% of the surveys that they started. Similarly, they completed around 90% of the BOD and EOD prompts over the first 6 months. With observations over 17,860 participant-days among the 150 participants, they initiated a survey to report an average of 1.48 lapses per week. When participants initiated a survey to report a temptation or lapse, they completed 98.44% (5055/5135) of those surveys, and they reported that they had a lapse in 43.80% (2214/5055) of the self-initiated surveys they completed. During the end-of-day surveys, participants stated that they did not report one or more temptations or lapses on 22.81% (5356/23,486) of the days.

**Table 2:** Adherence to ecological momentary assessment prompts by type of prompt during the first 6 and second 6 months of the study (N=150).

Prompt type		Completed	Abandoned^a^	Missed	Total
**First 6 months**					
	Random	66,978 (88.26%)	316 (0.42%)	8594 (11.32%)	75,888
	Event	5055 (98.44%)	80 (1.56%)	N/A^b^	5135
	BOD^c^	23,411 (90.29%)	143 (0.55%)	2375 (9.16%)	25,929
	EOD^d^	23,343 (89.75%)	132 (0.51%)	2535 (9.75%)	26,010
**Second 6 months**					
	Random	63,349 (85.10%)	244 (0.33%)	10,840 (14.56%)	74,443
	Event	2294 (98.75%)	29 (1.25%)	N/A	2323
	BOD	20,227 (85.61%)	131 (0.55%)	3269 (13.84%)	23,627
	EOD	20,308 (85.53%)	114 (0.48%)	3323 (13.99%)	23,745

^a^Abandoned means participant began a self-initiated report but discontinued before completing the items.

^b^N/A: not applicable.

^c^BOD: beginning of the day.

^d^EOD: end of the day.

As Table 3 reports, there was considerable variability among participants in the percentage completion of EMA prompts; note that differences in percentages reported in Table 3 are different from those reported in Table 2, since the means reported in Table 3 give equal weight to each participant, while the percentages in Table 2 give equal weight to each prompt. The medians are all greater than their respective means, since the distribution of percentage completed prompts is skewed to the left. While the range in completion rates for random assessments was quite wide, all but 1 participant completed half, and 80.7% (121/150) completed more than 80% of the random assessments. Variability in numbers of self-reports was especially high, with numbers of self-reports ranging from 0 to 567; only 1 participant never initiated a self-report. Participants were less adherent to daily weigh-ins and diet reporting using Lose It! than with the EMA assessments, and showed greater variability in the former than in the latter.

**Table 3:** Variability among participants in percentages of completed assessments by type and numbers of entered self-assessments (event contingent).

Assessment type	Mean	Median	SD	Range
Random	86.5%	88.7%	10.7%	16.7%-99.5%
Event	49.7	23	83.3	0-567
BOD^a^	87.6%	91.4%	12.4%	15.4%-100.0%
EOD^b^	87.3%	92.3%	13.0%	14.0%-100.0%
Weigh-ins	68.9%	76.9%	24.6%	4.1%-98.3%
Diet reporting	70.5%	78.3%	27.4%	4.4%-100.0%

^a^BOD: beginning of the day.

^b^EOD: end of the day.

The biggest challenge that we encountered was convincing participants to self-report episodes of temptation or lapses. This topic was raised with participants throughout the study. Some typical responses were “I am constantly tempted to eat what I shouldn’t so I would be initiating these surveys all day;” “When I am tempted, I need to walk away and forget it as soon as possible. The last thing I want to do is take time to report it;” or “I forget to report a temptation or lapse.”

## Discussion

We engaged in multiple phases of preparation and implementation to conduct a longitudinal observational study employing daily EMA prompts for 12 months. At the time of study launch, we used what was considered novel technological, methodological, and statistical means to address the critical issue of lapse and relapse after intentional weight loss, a significant clinical and public health issue that has been problematic in health care for over 30 years.

A major part of our preliminary studies was focused on development of the supportive infrastructure and refinement of the EMA assessments. In this process we learned a great deal. Primarily, if one wishes to have the EMA assessment on the phone and prevent any issues with connectivity with cell phone towers and potential loss of data, it is not possible to use a phone with an iOS operating system. Other investigators have handled this by providing their participant with a phone for EMA data collection, but the phone is locked for other uses [47]. Because of the duration of our study, and having learned that participants do not wish to carry 2 devices, we chose to provide participants a personal smartphone that accommodated the programming for EMA and that they could use for all purposes. This also prevented the loss of data when the person traveled, which likely has not been a major issue in the other EMA studies because of their shorter duration. However, today, many researchers are avoiding the issue of phone operating system compatibility by having the EMA program be Web based. Ehlers et al [48] demonstrated success in a 14-day pilot study with a low-cost approach that used participants’ mobile phones, text messaging, and mobile Internet to explore daily relationships between self-worth and physical activity in middle-aged women. Fanning et al [49] used a similar approach in a 7-day study of college-aged adults examining mind wandering.

Other salient lessons learned pertained to the maximum frequency of random EMA prompts that participants tolerated. We delivered up to 5 random prompts per day plus the BOD and EOD, and also asked participant to initiate event-contingent surveys. Another investigator prompted participants for 6 semirandom prompts per day plus an EOD survey, but that study was 14 days in duration [12]. Event-contingent completion has not been reported often but is essential if one is studying a time-varying emotion such as urge to smoke or to eat [4,11,20]. Important future work needs to examine whether the frequency of random prompts affects adherence to completion of other assessments.

Finally, we knew in the pilot studies and in EMPOWER that it was critical to ensure that all participants were receiving the prompts and the data were coming into the server. The overarching lesson learned was that, while parts of the study were run by programmed algorithms, careful oversight and monitoring were necessary to ensure that all components were functioning as intended. Staff members who filled other roles in the study participated in this oversight, similar to overseeing adherence to the protocol in a randomized clinical trial. Also, staff members were prepared to assist participants with troubleshooting the phone or EMA, which likely improved participants’ engagement, as they knew assistance was available and used it.

The length of previous EMA studies has ranged from 4 days [8] to 6 months [50]. As might be expected, retention is related to duration; for example, Stefano et al [51] reported on a study using smartphones to assess participants’ body monitoring behavior for 5 days and reported that only 1 person withdrew, resulting in 95.6% retention. Other EMA studies were conducted for 7 days [52], 14 days [48], and 1 month [53] and reported retention rates of 90.7%, 75%, and 66%, respectively. Inada et al [50] reported a 77.8% retention rate in their 6-month study. Gidlow et al [54] reported on a 12-week EMA study that was focused on stress among 153 healthy working adults that achieved a retention rate of 89.5%, confirming that, similar to our study, high rates of retention can be achieved in longer studies. Several studies that ranged from 4 [55-57] to 8 [58] to 18 weeks [59] did not report retention rates.

Adherence to responding to the EMA surveys, crucial for gaining insight into the targeted behaviors, also varies across studies, modes of survey administration, and types of EMA sampling used. Goldschmidt et al [12] used handheld computers to collect EMA data after each binge-eating episode plus 6 semirandom prompts per day and a before bedtime survey for 14 days. Participants responded to 86% of the semirandom prompts, while 84% of the bedtime recordings were completed. Zenk et al [13] used a smartphone and reported that 68.9% of the 35 random signal-contingent surveys were completed in a 7-day study about snack food intake.

Many studies use event-contingent sampling, which entails asking a participant to initiate an EMA survey to report an event, such as a binge-eating episode, consumption of snack food, or, as in our study, a temptation to eat a food that was not part of their dietary plan or the actual consumption of the food, which represented a lapse. It is not possible to report adherence rates to these self-initiated reports, only the frequency. Participants in Goldschmidt and colleague’s study reported 7.8 (6.5) binges and 11.1 (9.6) purges over the 12-day period [12]. McKee et al used only event-contingent sampling in their 7-day study of lapses among 80 adults who reported they were dieting and found that participants reported 898 instances of dietary temptation, or an average of 11.2 temptations per person over the 7 days [11]. These rates are significantly higher than those we observed in our study, which may be due to the 12-month duration of our study and that our participants were actively engaged in a weight loss intervention.

Our study was unique in its duration and 12-month retention rate of 87%. To date, no other study, to our knowledge, has assessed eating behaviors for as long as 12 months related to temptation, lapses, and relapse during a weight loss intervention. Our study contributes to the literature on EMA in describing the development and refinement of EMA items and the challenges and successes of developing the supporting infrastructure for a long-term EMA study. Additionally, our study adds information on strategies to enhance adherence in reporting episodes of slips and lapses through EMA.

## Abbreviations

BOD: beginning of the day
EMA: ecological momentary assessment
EOD: end of the day
PDA: personal digital assistant
SMART: Self-Monitoring And Recording using Technology
